# A Rare Complication of Perforated Appendicitis: A Case of Necrotizing Fasciitis

**DOI:** 10.7759/cureus.29679

**Published:** 2022-09-28

**Authors:** Vugar Suleimanov, Fatimah H Alhanabi, Fatima H Al Saeed, Hebah A Aldrazi, Hebatallah A Fagir

**Affiliations:** 1 Surgery, Jubail General Hospital, Jubail, SAU; 2 School of Medicine, Imam Abdulrahman Bin Faisal University, Dammam, SAU; 3 School of Medicine, Dali University, Yunnan, CHN

**Keywords:** diabetes insipidus, hearing impairment, clindamycin, omental infarction, perforation, necrotizing fasciitis, acute appendicitis

## Abstract

Acute appendicitis is considered one of the most common surgical emergencies with low morbidity and mortality. However, delay in the diagnosis may lead to perforation of the appendix. Hence, complications may arise, including necrotizing fasciitis, a rare complication of a perforated appendix. We present a case of perforated appendicitis complicated by necrotizing fasciitis leading to rapid deterioration.

A 75-year-old male patient presented to our emergency room with a three-day history of right lower quadrant abdominal pain and abdominal distention. On admission, computed tomography (CT) scan showed a perforated appendix and peri-appendicular abscess. An exploratory laparotomy was performed. The appendix was resected and the wound closed. The patient was recovering well and tolerating food after the procedure. However, the patient developed progressive erythema/swelling over the right flank with new-onset leukocytosis. The diagnosis of necrotizing fasciitis was suspected and confirmed by careful evaluation and laboratory/radiological tests. Antibiotics were changed to clindamycin and piperacillin/tazobactam, and the patient was taken back to the operation room (OR) for surgical debridement. Postoperatively, the patient was shifted to the intensive care unit (ICU). He developed hearing impairment, which improved after the cessation of clindamycin. He was discharged in good condition after three weeks of hospital stay.

This case report highlights the importance of maintaining a high index of suspicion for necrotizing soft tissue infection in immunocompromised patients with perforated appendicitis and being cautious when prescribing clindamycin to patients at risk of hearing loss.

## Introduction

Acute appendicitis is one of the most common causes of a surgical emergency for acute abdomen in children and young adults. Early diagnosis and management are crucial to improve morbidity and mortality [1]. The differential diagnosis of acute appendicitis includes a wide variety of medical and surgical conditions, which are well described in the Ergenç et al. study [2]. Ergenç et al. reported that diverticular disease of the appendix (DDA) has a similar presentation to acute appendicitis, and it is considered a rare pathology of the appendix [2].

Management of acute appendicitis depends on the severity of the presentation. Complicated appendicitis is usually treated by antibiotics if there is no abscess formation and the patient remains stable or by percutaneous drainage in addition to antibiotics if there is abscess formation, deferring appendectomy for four to six weeks [3,4]. Failure of response to antibiotics within 24 to 48 hours warrants surgical intervention. However, managing complicated appendicitis is a debatable subject. Some surgeons prefer immediate surgical intervention in case of phlegmon or abscesses [4,5]. On the other hand, initiation of conservative management prior to surgery is the preferred option for other surgeons [4,5]. Appendicitis in the elderly usually calls for a search for an underlying sinister cause.

Acute appendicitis carries a very low overall mortality of 0.27% if managed early [6]. A missed diagnosis of such cases is common in elderly patients, leading to numerous complications, including perforation of the appendix, abscess formation, peritonitis, and sepsis [6]. Necrotizing fasciitis is a necrotic infection of the fascia and subcutaneous tissues, characterized by rapid spread leading to sepsis [1]. Abdominal wall necrotizing fasciitis is a rare complication of a perforated appendix [7]. Moreover, it alone carries a 13.6% mortality [1]. On the other hand, a complicated perorated appendix with necrotizing fasciitis has a higher mortality rate, reaching 30-38% [8]. Early aggressive surgical debridement after diagnosis must take place to avoid systemic sepsis and enhance patient survival [1].

Here, we present a case of a rare complication, necrotizing fasciitis of the abdominal wall secondary to perforating appendicitis. The case demonstrates the importance of maintaining a high index of suspicion for necrotizing fasciitis in complicated appendicitis in elderly immunocompromised patients.

## Case presentation

A 75-year-old male patient, a known case of uncontrolled diabetes mellitus type two on oral hypoglycemic agents and hypertension, presented to the emergency department of our hospital on July 17, 2021, complaining of right lower quadrant pain of three days duration. The pain was associated with nausea, anorexia, and vomiting. There was a positive history of constipation and obstipation of one-day duration.

Upon physical examination, the patient was not in distress. His vital signs on presentation showed a temperature of 37.1 C, pulse rate of 91 beats per minute, blood pressure of 139/77 mmHg, O2 saturation of 99%, and respiratory rate of 18 breaths per minute. Labs were normal, except for a high white blood cell (WBC) count (13.9x10^3^ uL) and high random blood sugar (RBS) of 343.8 mg/dL. Abdominal examination revealed abdominal distention and a tender mass of 10 x10 cm over the right iliac fossa, with no changes in the overlying skin.

**Figure 1:**
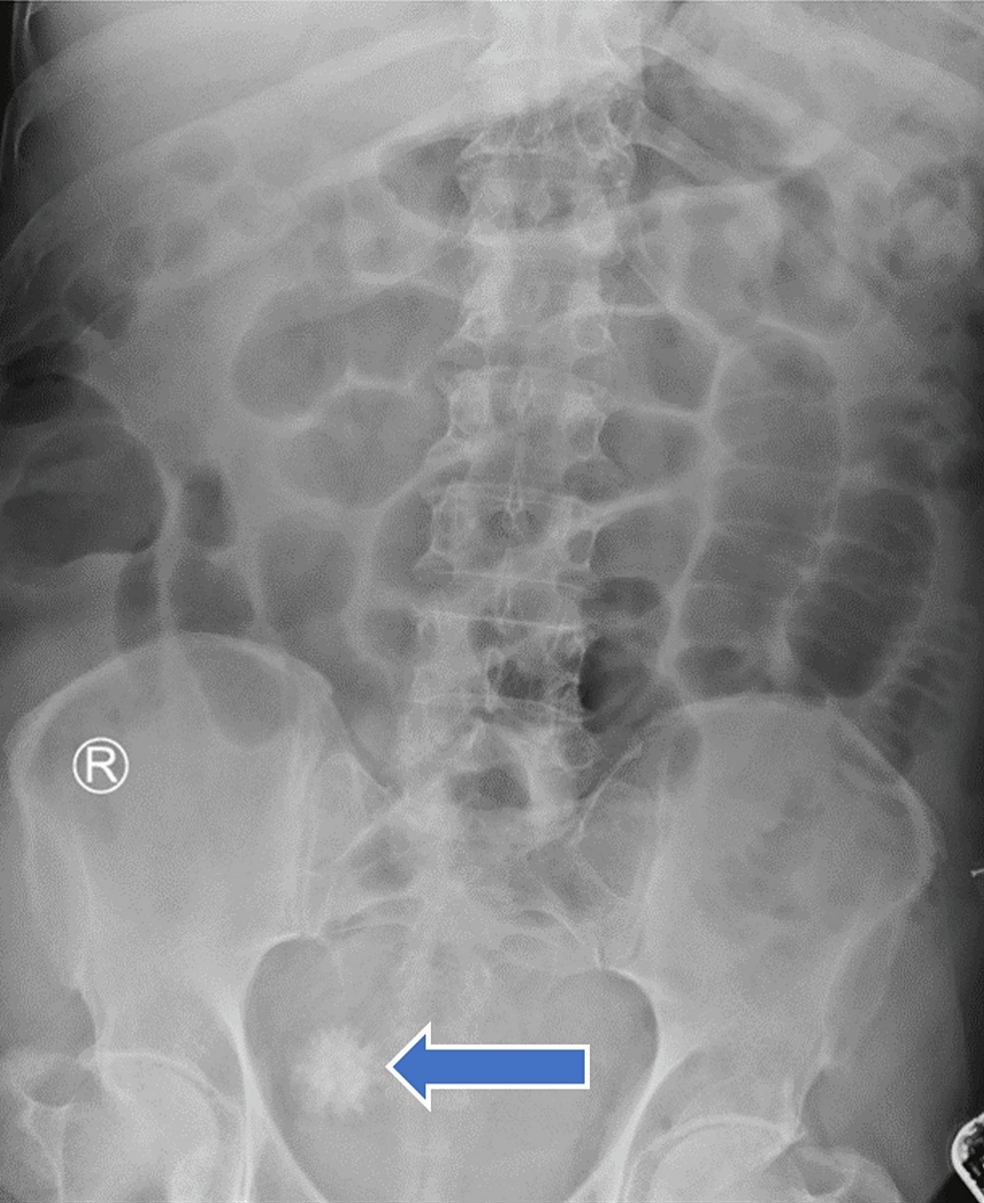
Plain abdominal X-ray showing dilated bowel loops The blue arrow indicates a radiopaque shadow in the pelvis, suggestive of a bladder stone.

**Figure 2:**
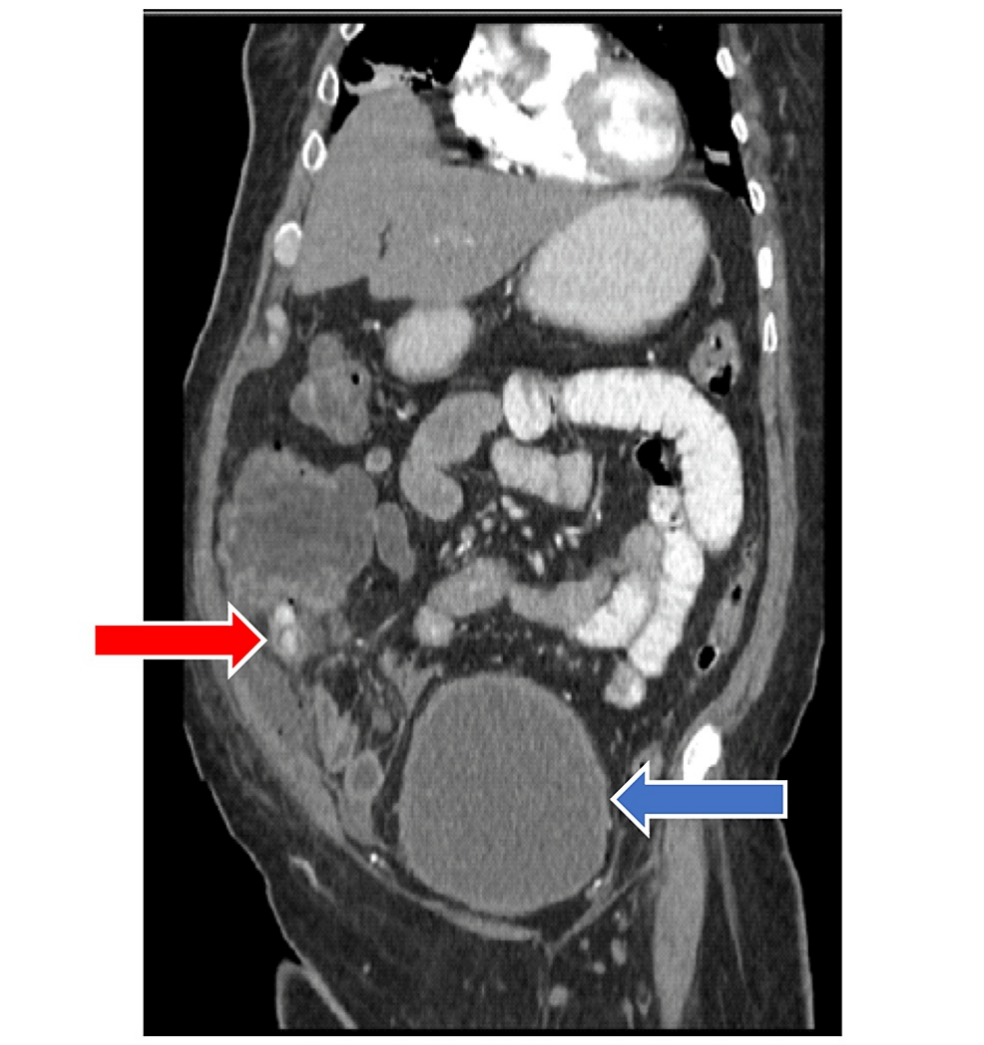
Sagittal view of an abdominal CT scan, showing a swollen appendix with two fecoliths (red arrow) and a large, thick-walled bladder (blue arrow)

**Figure 3:**
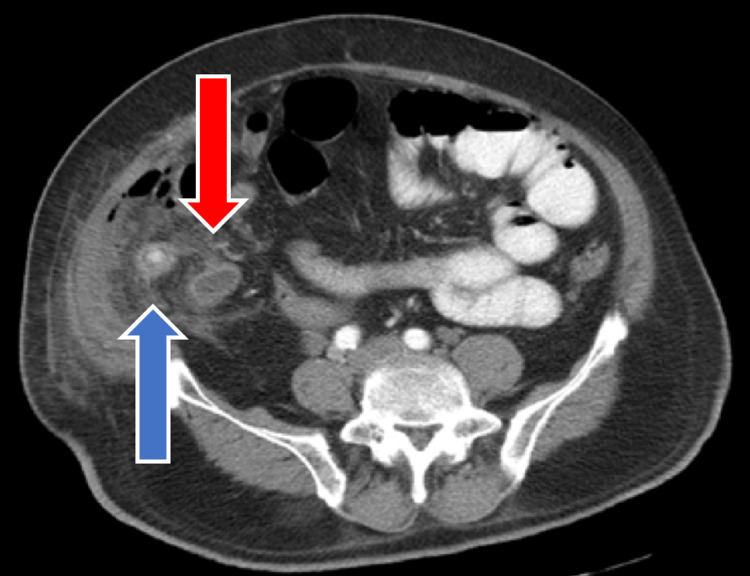
Axial view of abdominal CT shows swollen appendix with fecolith in the lumen, surrounded by an inflammatory mass (blue arrow) and a pocket of pus (red arrow)

**Figure 4:**
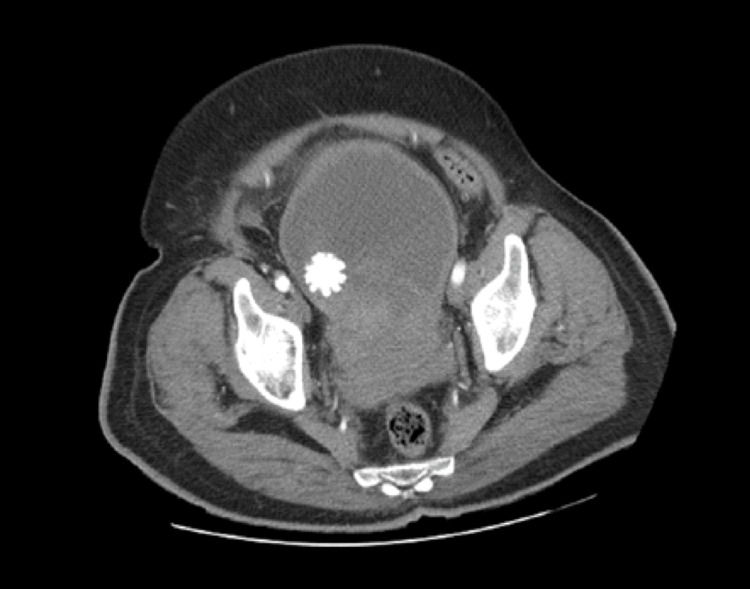
Axial view of abdominal CT shows an incidental finding of a bladder stone (blue arrow)

An abdominal X-ray showed dilated bowel loops with suspicion of a bladder stone (Figure 1). An abdominal CT scan showed a perforated appendix, peri-appendicular abscess, two large fecoliths at the base of the appendix (Figures 2, 3), dilated bladder with thickened wall, a bladder stone (Figures 2, 4), and a very large prostate. Additionally, swollen abdominal wall muscles were noticed on the right side (Figure 3).

After initial resuscitation by intravenous antibiotics, insulin, and venous thromboembolism prophylaxis, the patient was taken to the OR. Exploratory laparotomy was performed through a lower midline incision. A foul-smelling appendicular abscess was found. Additionally, perforation at the base of the appendix, two large fecoliths, and infarction of the omentum overlying the mass were noted. A trabeculated, thickened-wall bladder was also observed. Appendectomy and partial omentectomy were performed and a swab was taken for culture and sensitivity while the remaining omentum was wrapped around the cecum. Abdominal lavage and drainage were carried out, and the wound was closed after leaving a drain in the right iliac fossa. The swab culture grew Streptococcus anginosus, Escherichia coli, and Bacteroides fragilis. Moreover, antimicrobial susceptibility testing showed sensitivity to piperacillin/tazobactam and clindamycin.

Postoperatively, the patient had an uneventful recovery, where he started to tolerate oral intake and started to ambulate. However, edema and erythema over the right iliac fossa and right flank were observed, which was increasing in size gradually. On the fourth postoperative day, those manifestations worsened, and investigations revealed a white blood cell count of 19.3x10^3^ uL with 87% of neutrophils. CT scan was repeated, which showed significant abdominal wall edema. The laboratory risk indicator for necrotizing fasciitis (LRINEC) score was 10, and a diagnosis of necrotizing fasciitis of the abdominal wall was considered. Aspiration of the erythematous area was performed, revealing murky dishwater-like fluid, which was highly suggestive of necrotizing fasciitis. The patient was informed about the need for debridement, and informed consent was obtained.

**Figure 5:**
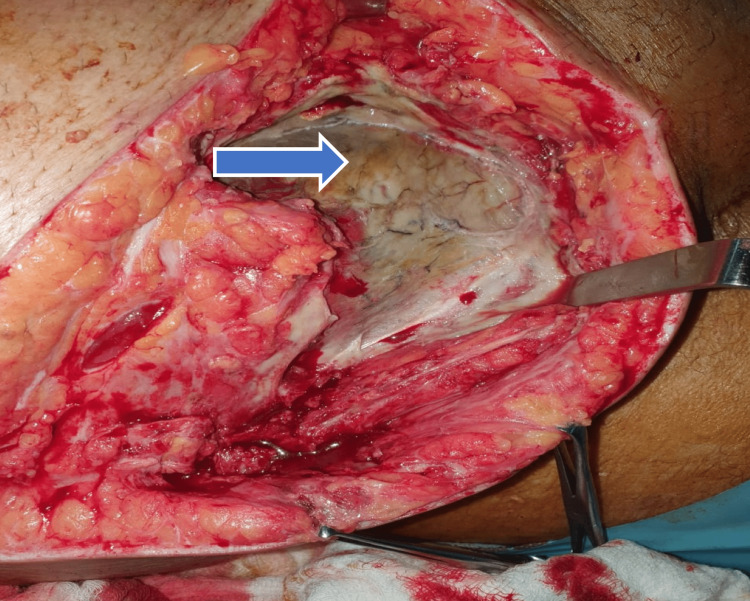
Necrotic abdominal wall muscles (external oblique, transverse abdominis, and internal oblique) and necrotic fascia were removed, exposing necrotic peritoneum (blue arrow)

**Figure 6:**
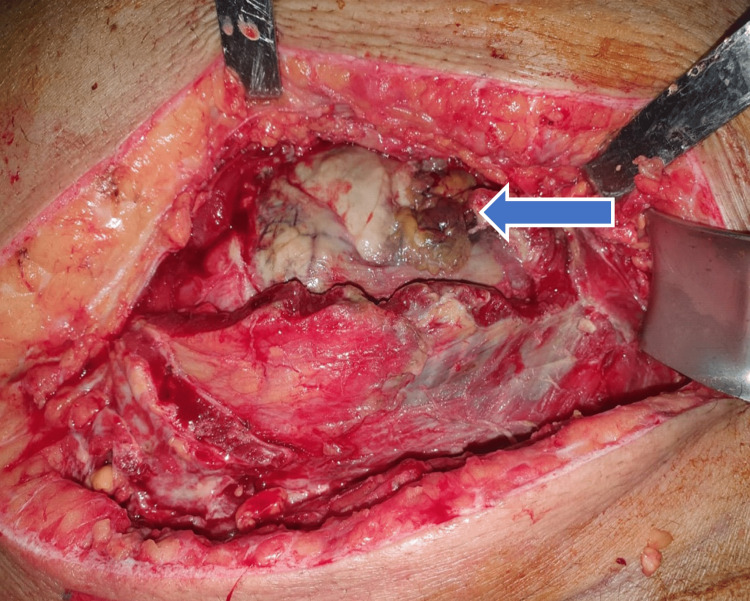
Necrotic peritoneum partially removed, revealing omentum covering the cecum (blue arrow).

Antibiotics were changed to piperacillin/tazobactam 3.375 g every six hours intravenous (IV) and IV clindamycin 900 mg every eight hours (he was taking cefuroxime and metronidazole previously) and the patient was taken to the OR. During the surgery, a 10 cm incision was made over the right iliac fossa, revealing necrotic subcutaneous tissues, fascia, muscles, and peritoneum (Figures 5, 6). Thereafter, a thorough debridement was performed down to the bleeding tissues.

The patient was shifted to the ICU. Pus culture grew Streptococcus anginosus, Escherichia coli, and Anaerobic bacteria. The patient was kept on a mechanical ventilator. Acid-base balance, fluid and electrolyte correction, and blood glucose control were all addressed. Nasogastric tube feeding was continued and well-tolerated. Thereafter, two additional debridements were performed in the OR on the fourth, fifth, and sixth days of the presentation. On the eighth day of presentation, the patient was extubated, and oral feeding was introduced.

It was noted that the patient was having an unusually high urine output, exceeding five liters per 24 hours, associated with hypernatremia, low urine osmolality, and increased thirst. Diabetes insipidus was a likely consideration and the patient responded well to nasal administration of desmopressin (DDAVP). A few days later, the patient was shifted to the floor, where wound care and antibiotics were continued.

While on the floor, the patient started to display hearing impairment, although he had normal hearing upon presentation. Significant hearing impairment was also noted by all personnel caring for him and his relatives. From the list of medications, the patient was receiving, only clindamycin could have contributed (very few reports in the literature showing vertigo and tinnitus as a side effect of clindamycin but no hearing loss) to this issue. IV clindamycin (900 mg three times daily) was started on the fifth day of presentation. The patient had a noticeable hearing impairment on the tenth day. Clindamycin was stopped on the 11th day (by this time the wound was improving and the spread of necrotizing fasciitis had stopped). Audiologist consultation was postponed till the patient's recovery for follow-up of hearing impairment. However, the patient's hearing improved on the 18th day. Upon discharge, the hearing was normalized, hence no audiogram was obtained.

Two weeks after the debridement of necrotizing fasciitis, the patient was taken to the OR, where secondary wound closure was performed after undermining skin flaps. The patient developed urinary retention due to a Foley catheter obstruction by a blood clot, which did not respond to catheter flushing. Changing the Foley catheter resolved the issue. After three weeks of hospital stay, the patient was discharged home in a stable condition, to be followed by a urologist in the outpatient department as a case of benign prostatic hyperplasia and urinary bladder stone. Additionally, the patient had a follow-up appointment in the general surgery clinic after one week of discharge, where the sutures were removed and good wound healing was noticed. The timeline of the major events that happened during the patient’s hospitalization is shown in Table 1.

**Table 1:** Timeline shows the patient's course from admission to discharge

Timeline																							
Surgical ward				Intensive care unit														Surgical ward					
Presentation to the emergency room, appendectomy	Wound care, antibiotics, and postoperative monitoring			Diagnosis of necrotizing fasciitis, surgical debridement, and mechanical ventilation.	Mechanical ventilation, nasogastric tube, repeated surgical debridement.		Mechanical ventilation, nasogastric tube.	Extubation and initiation of oral feeding.	Fluid and electrolyte correction, acid-base balance, glucose control, wound care, and IV antibiotics with oral feeding									Transferred to the male surgical ward.	Secondary wound closure.	Wound care and observation.			Discharge
0	1	2	3	4	5	6	7	8	9	10	11	12	13	14	15	16	17	18	19	20	21	22	23

## Discussion

A wide variety of common complications are associated with appendicitis, including perforation, peri-appendiceal abscess, peritonitis, bowel obstruction, and gangrene [9]. Nevertheless, a few rare complications of appendicitis are reported in the literature, including necrotizing fasciitis.

Necrotizing fasciitis is a rare but life-threatening complication of appendicitis caused by rapidly spreading necrotizing infection of the skin, subcutaneous tissues, and fascia, which might progress to sepsis, multiorgan failure, and death. Muscles are often spared [10]. Necrotizing fasciitis was first described by US Confederate Army Surgeon Joseph Jones, who described it as "hospital gangrene" [11]. The proper term "necrotizing fasciitis" was used for the first time by Wilson in 1952 [1]. Immunocompromised individuals including diabetics and cancer patients are at higher risk of developing necrotizing fasciitis. Necrotizing fasciitis has been classified into type one (polymicrobial), type two (monomicrobial), and type three (gas gangrene or clostridial myonecrosis) [1]. The most common causative organism is Streptococcus species, specifically, group A beta-hemolytic streptococci (GABHS). Nevertheless, a wide variety of other organisms were reported in the literature. This includes but is not limited to Staphylococcus aureus, Enterococcus species, Corynebacterium species, Lactobacillus, Escherichia coli, and Klebsiella. The majority of necrotizing fasciitis cases are polymicrobial, as evidenced by our case [12].

Recently, the frequency of necrotizing fasciitis has been rising due to the increasing number of immunocompromised patients with diabetes mellitus, cancer, alcoholism, vascular insufficiencies, organ transplants, HIV infection, or neutropenia. Our case was type one necrotizing fasciitis, which was a complication of perforated appendicitis. Early detection and management of necrotizing fasciitis are crucial for a better prognosis. Although the necrotizing fasciitis diagnosis is challenging, the LRINEC score is a helpful tool for the early diagnosis of necrotizing fasciitis. The score is based on laboratory data, including white blood cell count, glucose, creatinine, hemoglobin, sodium, and C-reactive protein. A positive score is achieved when it reaches six or more, thus it rises the suspicion of necrotizing fasciitis [13]. Rapid recognition is notoriously difficult, as it can be mistakenly diagnosed as cellulitis initially, which presents as edema and erythema [1]. A highly suggestive sign of necrotizing fasciitis is the presence of soft tissue crepitus. However, the absence of soft tissue crepitus does not rule out the diagnosis, as supported by our case [12]. Delay in diagnosis and management usually leads to the spread of infection along the fascial planes, causing widespread necrosis and sepsis in a short period [1].

Necrotizing fasciitis almost always complicates perforated retrocecal appendix due to delayed diagnosis and treatment [1]. But in our case, the perforated appendix was not retrocecal, it was in the usual place though the abscess was in contact with the anterior abdominal wall, hence the spread of infection to the abdominal wall took place. Edema of the abdominal wall was present at presentation and was seen on CT too, but it was not enough to make a diagnosis of necrotizing fasciitis on presentation. Besides, edema of surrounding tissues is almost always present when there is such an inflammatory process. Another interesting observation in our case was the development of a new onset hearing impairment, which we attributed to Clindamycin. Amazingly, the patient’s hearing was completely restored after a few days of cessation of Clindamycin. We did a literature search about this association, it seems that only a few case reports found such an association. Scissors et al. reported a case of a 14-year-old boy who was diagnosed with acne vulgaris. The patient was treated with topical clindamycin and benzoyl peroxide. Thereafter, he developed unilateral tinnitus and mixed hearing loss. He concluded that clindamycin has a role in the development of new complaints [14]. Our patient had one more unusual complication - diabetes insipidus, which again was not reported in the literature as a complication of necrotizing fasciitis. We used intranasal DDAVP, which resulted in a complete resolution of the situation in a few days.

Partial omental necrosis observed in our case needs some attention too. Among hundreds of cases of acute appendicitis, we have never observed omental necrosis like in this case. Omentum was gangrenous even far from the abscess/mass, which can be viewed as a red flag, heralding the start of necrotizing fasciitis.

The take-home message of this case report is to keep a high index of suspicion toward the rare complications of appendicitis, especially in elderly and immunocompromised patients.

## Conclusions

This case demonstrates a rare life-threatening complication of a common surgical condition that is frequently missed in elderly and immunocompromised patients. We emphasize early identification and management of this group of patients with broad-spectrum antibiotics and timely surgical intervention with aggressive debridement. A high index of suspicion for rare complications must be kept in mind to avoid lethal outcomes. This is one of few case reports showing an association between clindamycin use and hearing impairment, which must be borne in mind when prescribing clindamycin to patients at risk of hearing loss.
